# Identification of the First Functional Toxin-Antitoxin System in *Streptomyces*

**DOI:** 10.1371/journal.pone.0032977

**Published:** 2012-03-14

**Authors:** Laura Sevillano, Margarita Díaz, Yoshihiro Yamaguchi, Masayori Inouye, Ramón I. Santamaría

**Affiliations:** 1 Instituto de Biología Funcional y Genómica/Departamento de Microbiología y Genética, Consejo Superior de Investigaciones Científicas, Universidad de Salamanca, Campus Miguel de Unamuno, Salamanca, Spain; 2 Department of Biochemistry, Center for Advanced Biotechnology and Medicine, Robert Wood Johnson Medical School, Piscataway, New Jersey, United States of America; Universite Libre de Bruxelles, Belgium

## Abstract

Toxin-antitoxin (TA) systems are widespread among the plasmids and genomes of bacteria and archaea. This work reports the first description of a functional TA system in *Streptomyces* that is identical in two species routinely used in the laboratory: *Streptomyces lividans* and *S. coelicolor*. The described system belongs to the YefM/YoeB family and has a considerable similarity to *Escherichia coli* YefM/YoeB (about 53% identity and 73% similarity). Lethal effect of the *S. lividans* putative toxin (YoeBsl) was observed when expressed alone in *E. coli* SC36 (MG1655 *ΔyefM*-*yoeB*). However, no toxicity was obtained when co-expression of the antitoxin and toxin (YefM/YoeBsl) was carried out. The toxic effect was also observed when the *yoeBsl* was cloned in multicopy in the wild-type *S. lividans* or in a single copy in a *S. lividans* mutant, in which this TA system had been deleted. The *S. lividans* YefM/YoeBsl complex, purified from *E. coli*, binds with high affinity to its own promoter region but not to other three random selected promoters from *Streptomyces*. *In vivo* experiments demonstrated that the expression of *yoeBsl* in *E. coli* blocks translation initiation processing mRNA at three bases downstream of the initiation codon after 2 minutes of induction. These results indicate that the mechanism of action is identical to that of YoeB from *E. coli*.

## Introduction

Toxin-antitoxin (TA) modules were originally identified as plasmid maintenance or stability modules [1]. Later, such modules were described as being very abundant in the genome of different bacteria and archaea [2]. The role of these systems in the genome is not clear; however, they have been reported to act as guardians against DNA lost, serve as protection against invading DNA, and involved in stress management either through programmed cell death of a wide part of the population or contributing to the origin of persister cells by inducing a dormant stage (stasis) that permit to the cells to be highly tolerant to antibiotics [2], [3].

Regarding the nature and action of the antitoxin, three types of TA systems -class I, II, and III- have been described, class II being the most abundant [4]. This class II of TA systems comprises two small proteins, which act as a toxin-antitoxin complex in which the toxin is inactivated by the antitoxin. The efficiency of these TA systems depends on a difference in lifespan between the toxin and antitoxin. While toxins are highly resistant to proteases, the lifespan of antitoxin is shorter than the toxin owing to their high susceptibility to protease activity. When the toxins are released from the complex they produce their toxic effect through different modes of action: acting as endoribonucleases, poisoning DNA gyrase, inhibiting translation initiation or elongation or inducing defects in cell wall synthesis [5], [6]. Mainly, antitoxin counteracts toxin activity by direct protein-protein interaction and also by repressing transcription of the TA system through interaction with palindromic sequences within the promoters. In this regulation of their own promoter, toxins act as co-repressors, cooperatively improving the DNA interaction. However, in the case of three-component systems, antitoxin and DNA-binding activities are encoded by two separated proteins [7], [8], [9].

TA systems have been studied mostly in Gram-negative bacteria and, as usual, the organism studied in greatest depth is *Escherichia coli*, in which at least 33 TA systems have been identified [10]. The availability of a large number of genomes and the use of bioinformatics tools have permitted the identification of a huge number of putative TA systems in different microorganisms [11]; these have been completed in later studies and are accessible at some servers such as: http://genoweb.univ-rennes1.fr/duals/RASTA-Bacteria/ [12] and http://bioinfo-mml.sjtu.edu.cn/TADB/ [13]. In regards to the structure of these proteins and their activities, up to 12 toxin super-families and 20 antitoxin super-families have been described and validated [9].

Our work started after the prediction of the existence of three putative TA loci in the chromosome of *Streptomyces coelicolor* by Pandey and Gerdes [11]. The present study is the first experimental demonstration of the functionality of one of these systems in *S. lividans*, and in *S. coelicolor*. The orthologous genes of both organisms are identical, even in the promoter region, and correspond to an operon that encodes the proteins identified under the NCBI accession numbers ZP_06531415 and ZP_06531416 in *S. lividans* and to the proteins encoded by the operon formed by the SCOs2235/2236 from *S. coelicolor*. The system shows considerable similarity to the YefM/YoeB system from *E. coli*, composed of the protein YefM, which is an unstable antitoxin, and YoeB, which is a stable toxin. *E. coli* YefM/yoeB form a heterotrimer (toxin∶antitoxin 1∶2) that inactivates the effect of the toxin [14]. Free toxin acts by inhibiting translation initiation by associating directly with the 50S ribosome subunit. In particular, it interacts with the A site, originating mRNA cleavage and releasing the 3′-end portion of the mRNA from the ribosome [15].

Here we show that the introduction of the *S. lividans* toxin gene (*yoeB_sl_*) in multicopy plasmids is toxic in *E. coli* SC36, in *S. lividans*, and in *S. coelicolor* wild types strains and that its effect is reversed by the co-expression of the antitoxin gene (*yefMsl*). *S. coelicolor* (*ΔyefM/YoeB*) and *S. lividans* (*ΔyefM/yoeB*) null mutants have greater sensitivity to the toxin expression than the wild-type strains and the expression of a single copy of the corresponding gene is lethal. Purification of the *S. lividans* antitoxin (YefMsl), toxin (YoeBsl), and the TA complex (YefM/YoeBsl) allowed us to observe the specific binding of this complex to the promoter region of the bicistronic operon. *In vivo* experiments demonstrated that the toxin YoeBsl acts inhibiting translation initiation by processing the mRNA at three bases downstream of the initiation codon. The data reported constitute the first experimental demonstration of the functionality of a TA system in *Streptomyces*.

## Results

### Identification of TA systems in *Streptomyces*

Three TA loci were proposed in the chromosome of *S. coelicolor* by sequence similarity [11]. One of them was classified as a relBE type (GI: 21220706, 21220707) and the other two as phd/doc type(s) (GI: 21218953, 21218954 and GI: 21224247, 21224248). Here we experimentally characterized the first one (GI: 21220706, 21220707), encoded by the SCOs2235/2236 (these were named *yefMsc* and *yoeBsc*, respectively). Identical gene sequences were present in *S. lividans*, and they were designated *yefMsl* and *yoeBsl* respectively. The *yefMsl* gene encodes the putative antitoxin YefMsl (orthologous to YefMsc) and *yoeBsl* encodes the putative YoeBsl toxin (orthologous to YoeBsc). This corresponds to an operon that encodes the proteins identified under the NCBI accession numbers ZP_06531415 and ZP_06531416 respectively.

Although the system *yefMsc-yoeBsc* was first recognized as a relBE system, actual studies permit classify it as a hybrid system on which YefMsc antitoxin shows more sequence homology with Phd superfamily and the YoeBsc toxin belongs to the ParE/RelE toxin superfamily. These hybrid associations are more common than originally thought [9].

**Figure 1:**
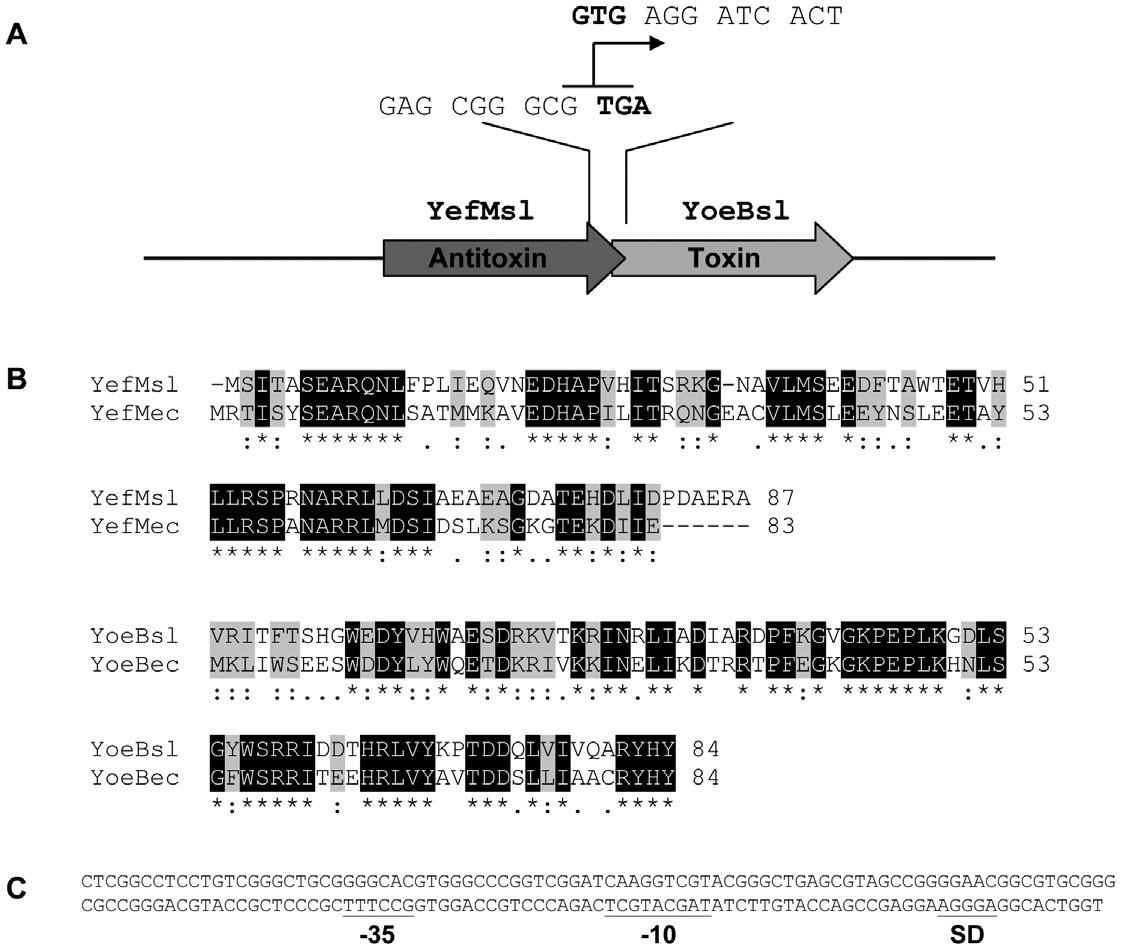
A) Schematic representation of the *S. lividans* Antitoxin-Toxin bicistronic operon. The overlap between the two genes is shown. The stop codon of the antitoxin and the start codon of the toxin are shown in bold. B) Clustal alignment of the predicted amino acid sequence of the putative *S. lividans* antitoxin (YefMsl) with *E. coli* YefM (YefMec) and the putative *S. lividans* toxin (YoeBsl) with *E. coli* YoeB (YoeBec). Identical amino acids are boxed in black and conservative amino acid substitutions are boxed in grey. Semi-conservative substitutions are shown with one dot. C) DNA sequence of the putative promoter of the *S. lividans* TA system. The putative -35 and -10 regions as well as the ribosome-binding site (SD) are underlined.

This locus is a bicistronic operon in which the upstream gene encodes the putative antitoxin of 9.7 kDa with a pI of 4.57 and the downstream one encodes the putative toxin of 9.9 kDa with a pI of 9.4. The last codon of the antitoxin gene overlaps with the first GTG codon of the toxin (Fig. 1A). Both proteins share clear identity with the YefM and YoeB proteins from *E. coli* (52% and 54% respectively) (Fig. 1B) and with other relBE-type TA systems from different microorganisms (data not shown). Upstream of the antitoxin gene, there is an intergenic region of 173 pb that may acts as the promoter of this system and it is identical in *S. coelicolor* and in *S. lividans*. Analysis of this region with BPROM identified the putative -35 and -10 boxes and a putative Shine-Dalgarno region (Fig. 1C).

### Overexpression of the *S. lividans* toxin gene is lethal in *E. coli*

**Figure 2:**
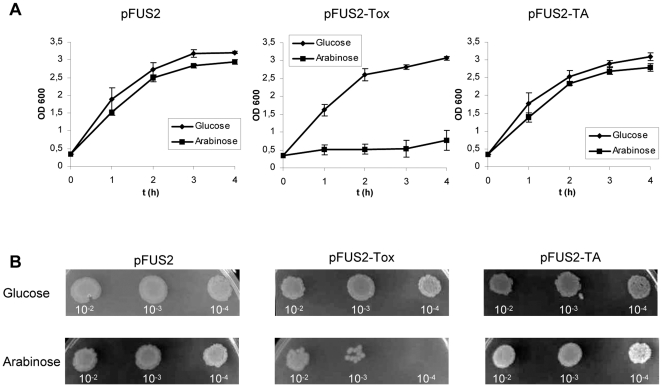
Effect of the overproduction of YoeBsl and YefM/YoeBsl complexes in *E. coli* SC36 (MG1655 Δ*yefM*-*yoeB*). A) Cells transformed with control plasmid (pFUS2), with the plasmid carrying *yoeBsl* (pFUS2-Tox), or with the plasmid carrying the *yefM/yoeBsl* (pFUS2-TA) were grown in liquid LB medium to mid-logarithmic phase. At this time (time zero), 0.2% glucose (diamond) was added to one half of each culture and 0.2% arabinose (square) to the other half. Cell growth was monitored measuring the OD_600_ of the cultures at different times. The means and standard deviation of three different experiments is presented. B) 5 µl of serial dilutions of the different cultures, taken one hour after protein induction, were inoculated in LB plates and incubated overnight at 37°C. The upper plates were inoculated with cells from media with glucose (repression), and the lower plates were inoculated with cells from media with arabinose (induction).

The effect of the overexpression of *yoeBsl* was studied in *E. coli* SC36 (Δ*yefM*-*yoeB* strain). PCR amplification of the *yoeBsl* gene from the *S. lividans* genome and cloning into the pFUS2 plasmid [16] provided the pFUS2-tox plasmid (Materials and Methods). In this plasmid, the *S. lividans* toxin gene is under the control of the arabinose-inducible P_BAD_ promoter. Plasmid pFUS2-tox was transferred into *E. coli* SC36, and cell growth was tested in the presence of glucose or arabinose (repressing and inducing conditions respectively). Normal cell growth was observed when the *E. coli* (pFUS2-tox) cells were cultivated in the presence of glucose. However, a strong reduction in cell growth was observed when the liquid cultures were shifted to an arabinose-containing medium (Fig. 2A). A reduction in the number of viable cells was also observed when the different cultures were inoculated on LB plates one hour after protein induction (Fig. 2B). In contrast, the growth of *E. coli* strains containing pFUS2 (empty plasmid) or pFUS2-TA (which carries the complete operon under the control of the P_BAD_ promoter) was normal and fairly similar in both liquid glucose- and arabinose-containing medium (Fig. 2A) and also on LB plates (Fig. 2B). The same result was obtained when a carboxy-His-tagged YoeBsl (pFUS2-ToxHis_6_) was produced in *E. coli* (data not shown).

These results demonstrated that the protein encoded by the *yoeBsl* gene is a potent toxin against *E. coli* cells and that the protein encoded by *yefMsl* can counteract this toxicity when it is expressed at the same time. Therefore, these observations indicate that this operon works as a typical TA locus in *E. coli*.

### Overexpression of the toxin gene is lethal in *S. lividans*

**Figure 3:**
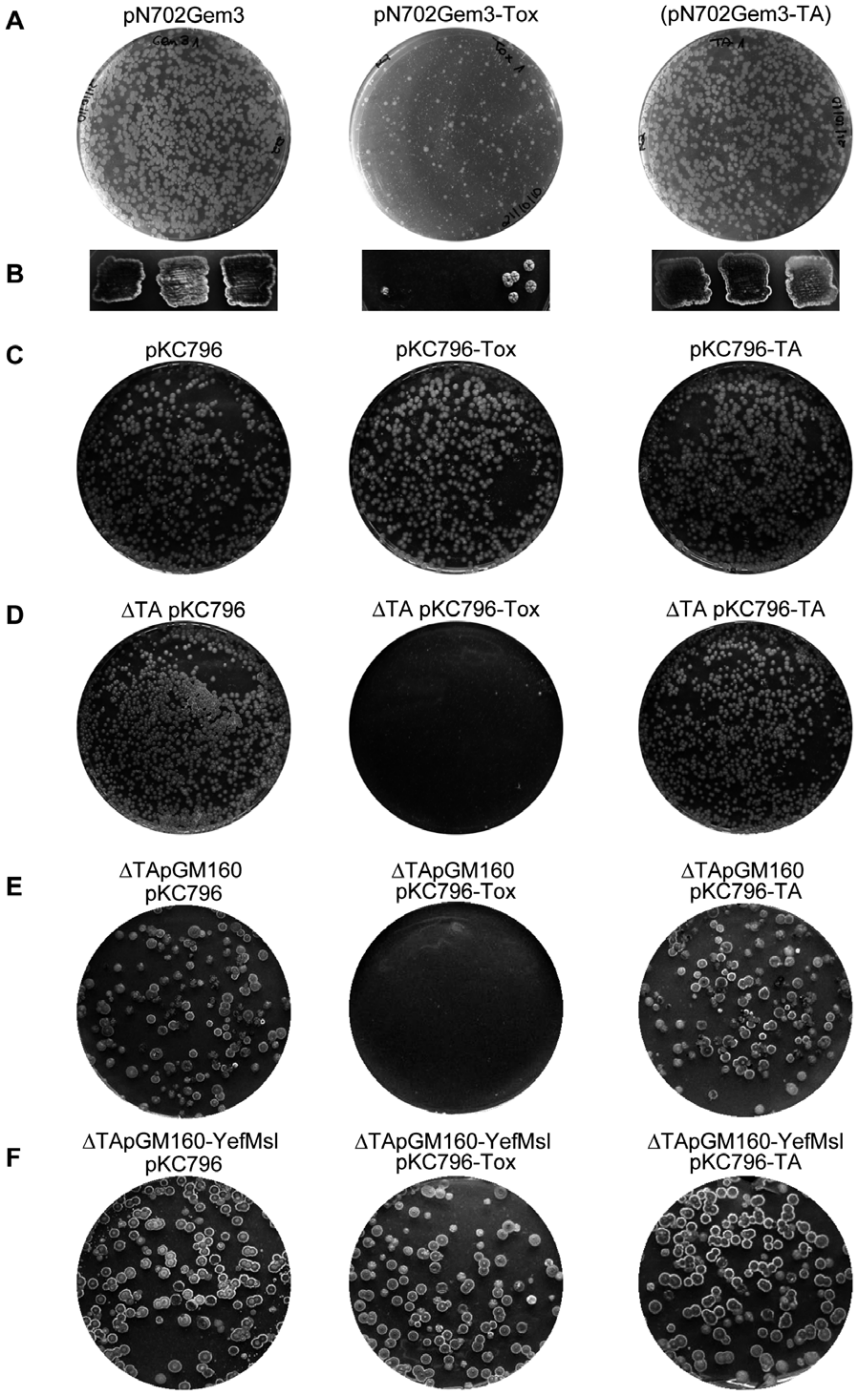
Effect of YoeBsl and YefM/YoeBsl complexes on the viability of the wild-type *S. lividans* (A, B, C), and on the *S. lividans * Δ**TA (**Δ***yefM/yoeBsl*** **) mutant (D, E, F).** A) R2YE agar plates showing the colonies obtained in transformations with the same DNA amount of empty multicopy vector (pN702Gem3), the plasmid carrying the *yoeBsl* gene (pN702Gem3-Tox) or the plasmid containing *yefM/yoeBsl* genes (pN702Gem3-TA). B) Viability of the colonies obtained in the transformation after streaking them onto R2YE media containing 1% xylose (inducing conditions of the *xysAp* promoter). C) R2YE agar plates showing the colonies resulted from the transformation of the *S. lividans* wild type strain with the same DNA amount of empty integrative vector (pKC796) or with this plasmid carrying the *yoeBsl* gene (pKC796-Tox) or the plasmid carrying the *yefM/yoeBsl* genes (pKC796-TA). D) As in C, but using the *S. lividans* ΔTA *(ΔyefM/yoeBsl)* mutant as host. E and F) Effect of coexpression of YefMsl and YoeBsl, from different compatible plasmids, in *S. lividans ΔyefM/yoeBsl* mutant. Protoplasts of *S. lividans ΔyefM/yoeBsl* mutant carrying the empty multicopy vector pGM160 (E) or the plasmid pGM160-yefMsl (F) were transformed with the same amount of integrative plasmid pKC796 or its derivatives pKC796-Tox or pKC796-TA and inoculated in R2YE plates. The presence of pGM160-yefMsl in this strain eliminates the lethality originated by pKC796-Tox.

The effect of overexpression of the putative toxin encoding gene *yoeBsl* was also studied by transforming wild-type *S. lividans* protoplasts with a multicopy plasmid (a pN702Gem3 derivative) that expressed *yoeBsl* gene under the control of the strong *Streptomyces* promoter *xysA*p [17]. The number of transformants obtained in the *S. lividans* wild-type strain with the multicopy plasmid pN702Gem3-Tox was very low in comparison with the number of transformants obtained when the same protoplasts were transformed with an identical amount of the empty plasmid (pN702Gem3) or with plasmid pN702Gem3-TA, bearing the complete operon. Also, the colonies obtained with the plasmid pN702Gem3-Tox were smaller in size indicating the toxicity of the YoeBsl protein due to its overexpression in *S. lividans* (Fig. 3A). In addition, the few colonies carrying the plasmid pN702Gem3-Tox hardly grew when were reinoculated on patches on plates of R2YE medium supplemented with 1% xylose (*xysAp*-inducer) suggesting that the accumulation of the toxin in these cells make them non viable (Fig. 3B). However, when *S. lividans* wild type protoplasts were transformed with an integrative plasmid pKC796-Tox (a pKC796 derivative that carries the *yoeBsl* gene under the control of the same promoter (*xysA*p) [17]) the number of colonies was similar to that obtained with the pKC796 empty plasmid or with the pKC796 derivative containing the complete operon (pKC796-TA) (Fig. 3C).

These results indicated that the toxicity of YoeBsl seemed to depend on the amount of protein accumulated in the cell and suggested that the expression of the endogenous single copy of the antitoxin encoding *yefMsl* gene in the genome could counteract the toxic effect of an extra copy introduced with pKC796-Tox, but not the higher amount originated by the multicopy plasmid pN702Gem3-Tox.

To test this hypothesis, a deletion of the *yefM/yoeBsl* operon was performed in *S. lividans* by means of REDIRECT technology (see Materials and Methods) and the resulting mutant (Δ*yefM*/*yoeBsl*) was used as a receptor for plasmids pKC796-Tox and the corresponding empty vector. The integration of a single copy of *yoeBsl* in this ΔTA strain (plasmid pKC796-Tox) was lethal and no transformants were obtained, while the number of colonies obtained with the control plasmids was similar to that obtained in the wild-type strain (Figure 3D). However, when the *S. lividans* Δ*yefM*/*yoeBsl* null mutant was transformed with a plasmid containing the antitoxin gene and then the *yoeBsl* gene was integrated in the genome with pKC796-Tox the number of colonies obtained was similar to that obtained with control plasmids (pKC796 and pKC796-TA). This assay demonstrates that the inhibition of colony formation induced by a single copy of the toxin is counteracted by the expression of the antitoxin gene (Figure 3E and 3F).

The same experiments were carried out with a *ΔyefM/yoeBsc* mutant of *S. coelicolor*, obtained also along this work, and identical results were obtained (data not shown).

All these results demonstrate that in fact, this TA system works as a typical TA system in *Streptomyces*.

### The *S. lividans* Toxin-Antitoxin complex interacts with its TA promoter

**Figure 4:**
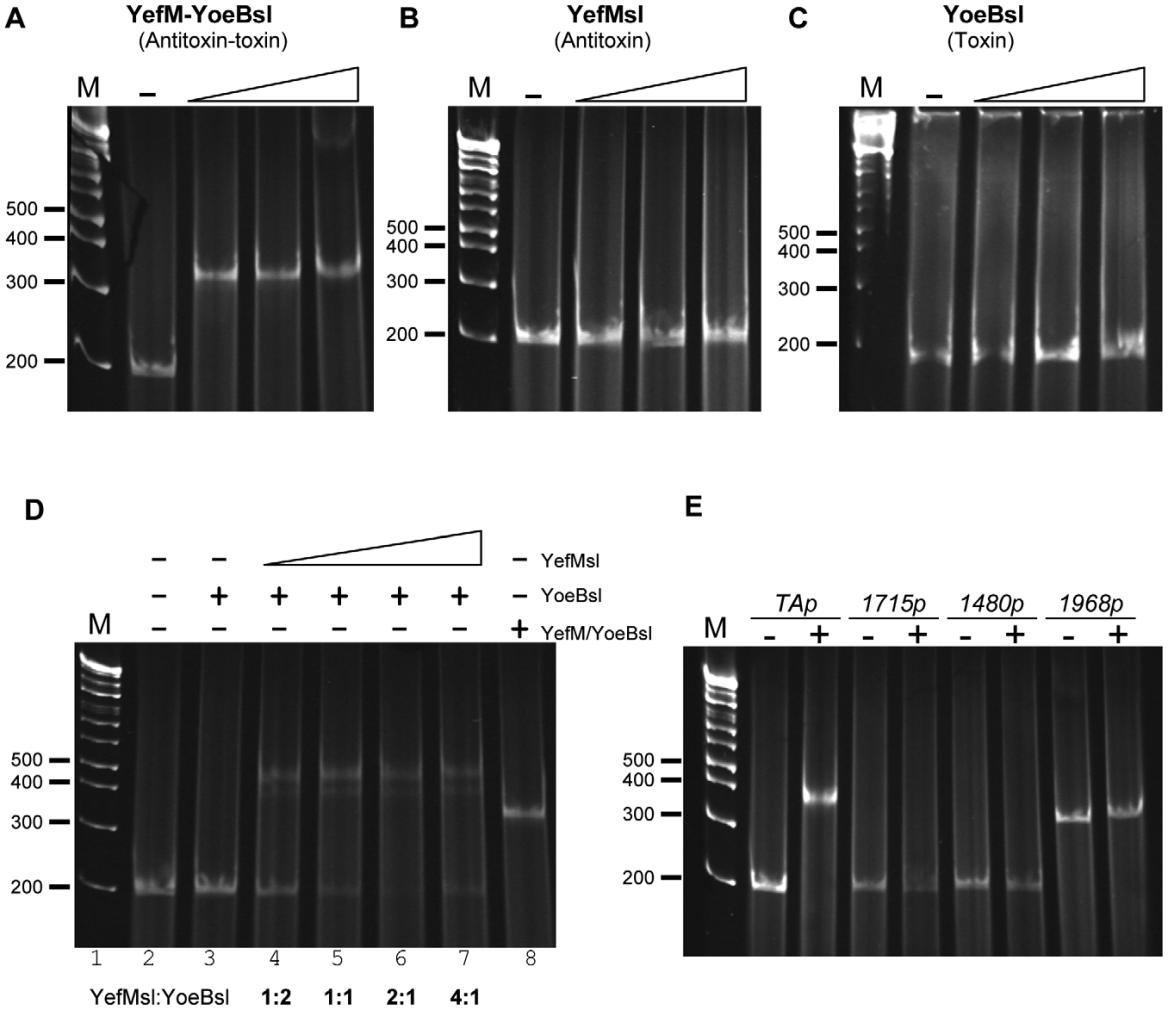
EMSA assays of the 173 bp intergenic region upstream of the *yefM/yoeBsl* operon with different proteins: (A) purified complex YefM/YoeB-His_6_sl (0, 1, 2 and 5 µM); (B) purified YefM-His_6_sl (0, 2, 4 and 8 µM); (C) Purified YoeB-His_6_sl (0, 1, 2, and 4 µM). D) EMSA assays with *in vitro* reconstituted YefM-His_6_/YoeB-His_6_sl complexes. The absence or presence of YoeB-His_6_sl, and/or YefM-His_6_sl is indicated by −/+ respectively. 2 µM of YoeB-His_6_sl were used (lanes 3–7), and mixed with increasing amounts (1, 2, 4 and 8 µM) of YefM-His_6_sl (lanes 4–7). 1 µM of natural purified YefM/YoeB-His_6_sl complex was used as a control (lane 8). E) EMSA assays of different promoters from *S. coelicolor* (the SCO number is indicated) with (+) or without (−) 1 µM of the purified complex YefM/YoeB-His_6_ sl.

The binding of toxin-antitoxin complexes to their promoters is the main way of regulation of the TA operon [18]. To study this, the *yefMsl* gene or the complete *yefM*/*yoeBsl* operon, were cloned in the *E. coli* expression vector pET22b under the control of the T7 promoter, obtaining pET22b-Anti and pET22b-TA respectively (Materials and Methods). These plasmids were introduced into *E. coli* BL21 (DE3) cells, and the antitoxin and the antitoxin-toxin complexes were purified. The toxin protein alone was purified from the antitoxin-toxin complexes as indicated in materials and methods. EMSA assays were used to analyze the interactions of *Streptomyces* TA complexes with the 173 bp double-stranded DNA corresponding to the intergenic region upstream of the antitoxin gene of *S. lividans*. The purified toxin-antitoxin complexes produced a single retardation band in the migration of the DNA corresponding to the TA promoter at a concentration of 1 µM (Figure 4A). However, no band-shift was detected, under the used conditions, when only the antitoxin (concentrations of 2–8 µM) or the toxin (concentrations of 1–4 µM) was used to bind the promoter (Figure 4B and 4C respectively). EMSA assays with the *in vitro* reconstituted YefM-His_6_/YoeB-His_6_sl complex were performed at different antitoxin/toxin ratios, and this permitted us to observe that the best ratio for DNA retardation was 2∶1 (Figure 4D). Two retardation bands were observed, one more intense than the other, and none of them migrated to the same level as the retardation band obtained with the natural-antitoxin-toxin complex purified from *E. coli* cells (Figure 4D lanes 4, 5, 6 and 7 *versus* lane 8) (see discussion).

No retardation was detected when three other *S. coelicolor* promoters, randomly selected (Materials and Methods), were used (Figure 4E). Thus, the interaction of the YefM/YoeBsl complex with the DNA of its promoter was specific and was correlated with the typical regulation of other TA systems described in different organisms.

### Purified YoeBsl inhibits protein synthesis on a Cell-free system

**Figure 5:**
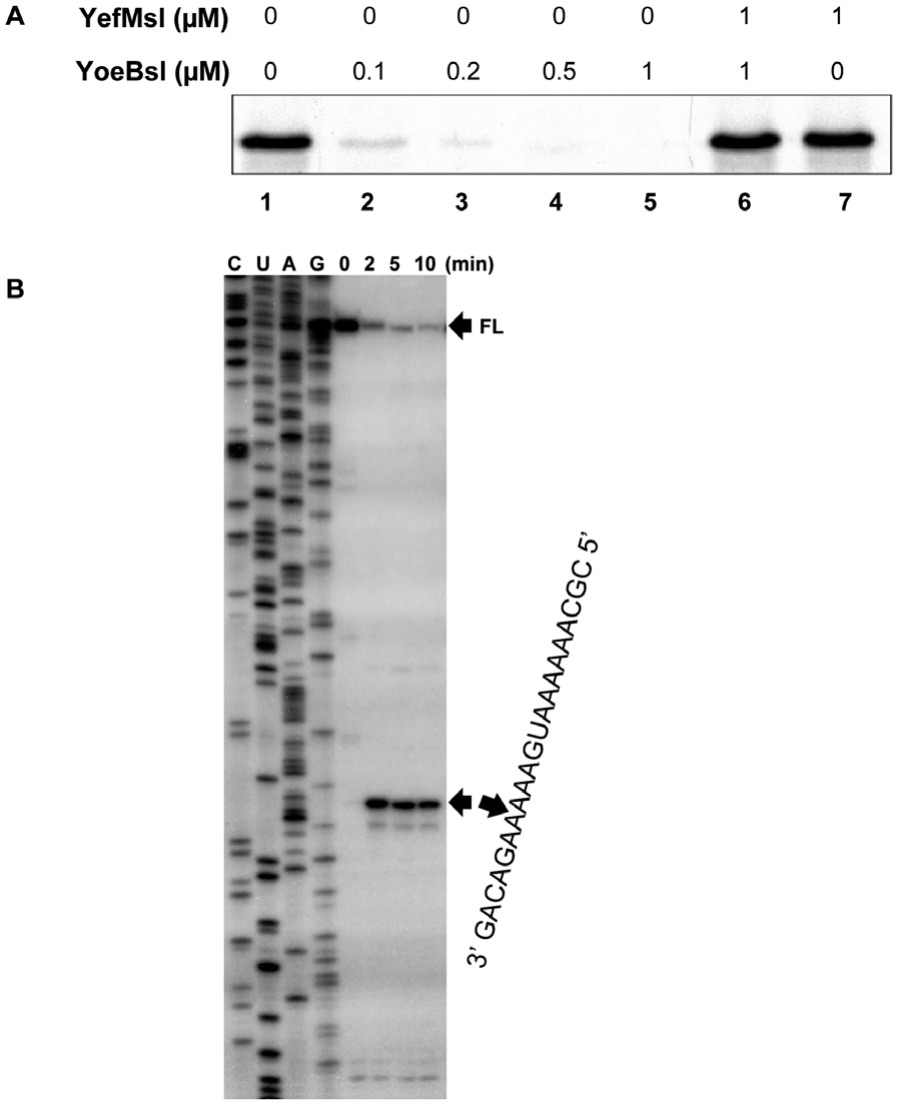
Effect of YoeBsl on protein synthesis in a cell-free system and Primer extension analysis of YoeBsl cleavage sites in the *ompA* mRNA *in vivo*. A) MazG protein synthesis was carried out using *E. coli* T7 S30 extract system for circular DNA (Promega) with pET11a-*mazG*. Lane 1, without YoeB-His_6_sl; lanes 2 to 5, 0.1, 0.2, 0.5 and 1 µM YoeB-His_6_sl were added, respectively; lane 6, 1 µM YoeB-His_6_sl plus 1 µM YefM-His_6_sl; and lane 7, 1 µM YefM-His_6_sl was added. B) Total RNA was prepared from *E. coli* BL21 cells harboring pFUS2-Tox at indicated time points before and after the induction of *yoeBsl* expression. The major cleavage site is indicated by an arrowhead on the right side of the gel. The sequence of the major cleavage site in the *ompA* mRNA is also shown on the right side of the gel. The sequence ladder for *ompA* was obtained using pCR®2.1-TOPO-*ompA* as template. The full-length RNA bands (FL) are indicated with an arrow.

The effect of purified YoeB-His_6_sl on cell-free protein synthesis was examined over MazG. The synthesis of MazG protein from plasmid pET11a-*mazG* was performed at 37°C for 30 min in the absence and presence of YoeB-His_6_sl using an *E. coli* T7 S30 extract system (Promega) (Fig. 5A). MazG synthesis was almost completely blocked at YoeB-His_6_sl concentrations of 0.5 µM or above. Similar inhibition was observed when purified YoeBec was added [15]. We then tested the effect of YefM-His_6_sl antitoxin on the YoeBsl-mediated inhibition of MazG synthesis and found that the addition of YefM-His_6_sl recovered MazG synthesis (Fig. 5A, lane 6).

### YoeBsl cleavages *ompA* mRNA *in vivo*

We next attempted to identify the cleavage sites of the *ompA* mRNA by primer extension experiments. For this purpose, total RNA was extracted from *E. coli* BL21(DE3) cells harboring pFUS2-Tox at different time intervals following induction of *yoeBsl* expression. The primer extension analyses of the *ompA* mRNA demonstrated that the distinct major bands exhibiting the specific cleavage sites in the mRNA appeared as early as 2 min after YoeBsl induction (Fig. 5B, lane 2). The major bands resulted from the cleavage of the mRNA at three bases downstream of the initiation codon, AUG, and most notably no other bands were observed in the regions between the 5′-end of the mRNA and the initiation codon indicating that YoeBsl may function only when it associates with the ribosomal translation machinary. These data showed that YoeBsl might primarily inhibit translation initiation, as shown with *E. coli* YoeBec [15].

## Discussion

The putative roles of TA systems located in genomes are not clear. Some of them have been implicated in programmed cell death under different stress conditions, such as MazE/F from *E. coli* [19], (and references therein) or in the fruiting body formation of *Myxococcus xanthus*, where a MazF homologue is involved in the regulation cascade controlled by the MrpC regulator [20]. Several models have been proposed to explain the persistence of these systems in genomes, where they may contribute to the stability of the genomes and plasmids, and might act as anti-addiction modules by preventing post-segregational killing [2], [19].

The abundance of these systems in different organisms has been detected thanks to the massive sequencing and the use of bioinformatics tools, which have shown that TA systems may represent 1.5% of the coding sequences in certain free-living microorganisms. In fact, this presence may be more abundant in some obligate intracellular species such as *Ricketsia bellii* OSU 85–389, where they represent 2.2% of its ORFs. However, TA systems are absent in other obligate intracellular species such as *Buchnera aphicola* or in different species of *Chlamydia*, suggesting their loss due to genome reduction [9]. Horizontal gene transfer associated with mobile genetic elements has been proposed to explain the presence of highly conserved orthologous in phylogenetically different organisms [2].

Only three TA systems had been proposed in *S. coelicolor* [11] when we began this work. However, recently, up to 24 TAs have been proposed [13], although in no case has their functionality been demonstrated. Here, the first study of the functionality of one of these putative TA systems of *Streptomyces* is described, corresponding to the SCOs2235/2236 of *S. coelicolor* and to the ZP_06531415/ZP_06531416 sequences of *S. lividans*. This system is similar to the YefM/YoeB system from *E. coli*, sharing about 53% identity and 73% similarity in both proteins. Even, two arginine residues highly conserved in the *E. coli* YefM orthologs (R10 and R31) [5] that are important in the direct interaction with the operator sequence are conserved in *Streptomyces* antitoxin in similar positions (R9 and R31). This observation suggests a putative horizontal transfer of this TA system between the ancestors of both organisms due that other randomly taken *S. coelicolor* and *S. lividans* genes from primary metabolism share only about 20–34% identity with their *E. coli* orthologous genes.

BLAST analysis of the proteins studied (YefM/YoeBsl) against 16 species of *Streptomyces* sequenced by the Broad Institute (http://www.broadinstitute.org/annotation/genome/streptomyces_group/GenomesIndex.html) confirmed the presence of YefM/YoeBsl orthologous in 7 of them and their absence in another 9 *Streptomyces* species. Surprisingly, some orthologous genes from different *Streptomyces* species have lower identity with the system studied than the YefM/YoeB system from *E. coli*. It is also of interest that two strains -*S. hygroscopicus* ACCT53653 and *Streptomyces* sp. C- each have two YefM/YoeB orthologous systems in their genome that are fairly similar (90% and 79% similarity, respectively) to the TA studied here, suggesting a putative total or partial duplication of this TA system. Interestingly, one of these putative TA systems in *S. hygroscopicus* ACCT53652 has a putative toxin (ZP_07297953) with only 52 amino acids, while *S. coelicolor* and *S. lividans* toxin has 84 amino acids.

The organization of the *yefM/yoeBsl* operon is similar to other bacterial TA modules in which the first gene encodes the antitoxin and overlaps the toxin gene in three nucleotides, supporting the existence of translational coupling between both genes. Upstream of the antitoxin gene, there is an intergenic region of 173 nucleotides that may act as a bidirectional promoter region and that is identical in *S. coelicolor* and *S. lividans*. This promoter has the TA operon on one side and the *glnE* gene that encodes the glutamate-ammonia-ligase adenylyltransferase on the other. EMSA assays demonstrated the high capacity of the YefM/YoeBsl complexes to bind this intergenic region. A divergent promoter is also present upstream of the *E. coli yefM/yoeB* system, but in this case the *hisL* gene is present on the other side of the promoter. Long and short palindromes with the core motif 5-TGTACA-3 are present in the *E. coli* promoter and have been described as the binding site for the YefM/YoeBec complexes [5], [21]. In the *Streptomyces yefM/yoeB* promoter there is a palindromic sequence 5′-TCGTACGA-3′ overlapping the putative -10 region and a downstream almost perfect palindrome 5′-TGTACC-3′ separated by a centre-to-centre distance of 12 bp that is the same distance between the palindromic sequences described in *E. coli*. Preliminary results indicate that these sequences form part of the binding sites of the YefM/YoeBsl complex (data not shown).

Deletion of the complete *yefM/yoeB* operon from *S. coelicolor* and from *S. lividans* originated strains with a slight retardation on sporulation when cultured on MSA medium but no other phenotypes were obviously different from the corresponding wild-type strains (data not shown). This “no-effect” has been described for simultaneous deletions of several TA systems from *E. coli* [22]. However, more detailed studies of these *E. coli* strains have later revealed differences in the capacity of this TA-deleted strain to produce biofilm. In particular, those authors observed that the *E. coli* antitoxin YefM clearly increased biofilm formation through an as yet unknown mechanism [23]. These results point to the need for a large number of tests aimed at identifying the true role of the *Streptomyces* TAs under laboratory conditions.

Overproduction of YoeBsl in the Δ*yefM*/*yoeB E. coli* SC36 strain originated a strong reduction in cell viability that was reversed by the co-expression of the YefMsl antitoxin. A similar lethal effect was observed when only one copy of the toxin gene was integrated into the chromosome of the *S. lividans*, or *S. coelicolor* Δ*yefM*/*yoeB* mutants, where no transformants were obtained. However, this toxic effect was not observed in *S. lividans* or *S. coelicolor* wild-type strains when an extra copy of the toxin gene was integrated in the genome, and the toxic effect was only detected when a multicopy plasmid was used to express the toxin gene. These results suggest that in these strains the endogenous antitoxin was able to block the lethal effect of an extra copy of the toxin gene but not enough when more copies of the gene were present.

*In vitro* experiments demonstrated that the His-tagged YoeB-His_6_sl and YefM-His_6_sl proteins are active in experiments of inhibition of protein synthesis. However, EMSA assays, demonstrated that *in vitro* reconstituted YefM-His_6_/YoeB-His_6_sl complex produced two retarded bands with the cognate promoter instead of one as it was obtained with the *S. lividans* TA complex purified from *E. coli*. These results suggest a different conformation of the reconstituted complex maybe due to the extra His_6_-tag present in the antitoxin carboxy terminus. Similar results have been described previously with the YefM/YoeB proteins from *E. coli* [21].

*In vivo* experiments with the protein YoeBsl demonstrated that its activity is identical to YoeBec processing *ompA* mRNA mainly at three bases downstream of the initiation codon. However, unlike YoeBec processing, where a significant amount of full-lengh *ompA* mRNA remains even 30 min after induction of the *E. coli* toxin [15], most of the full-lengh mRNA disappears after only two minutes of *yoeBsl* expression. This result suggests a higher mRNA processing activity of the YoeBsl compared to YoeBec.

## Materials and Methods

### Bacterial strains and growth conditions

The *E. coli* strains used were as follows: DH5α [24] for the cloning and isolation of plasmids; BL21 (DE3) (Stratagene) to express and purify proteins, and SC36 (MG1655 Δ*yefM*-*yoeB*) [25] to evaluate toxin toxicity. *E.coli* BW25113 (pIJ790), ET12567 (pUZ8002), and DH5α (pBT30) were used for gene replacement [26]. All strains were grown in Luria-Bertani (LB) liquid broth or on LB agar. All manipulations in *E. coli* were performed following standard procedures [24].

*S. coelicolor* M145, *S. lividans* 1326 and derivatives were grown on solid R2YE medium for transformation, on MSA medium for sporulation [27], and in liquid YES medium (1% yeast extract 10.3% sucrose [pH 7.2] supplemented with 0.5% glucose, 5 mM MgCl_2_ and 0.5% glycine) for collecting cells to make protoplasts. Liquid cultures were carried out in baffled flasks at 28°C and 200 rpm. All manipulations in *Streptomyces* were done as indicated by Kieser [27].

### Deletion of the *yefM/yoeB* operon in *S. coelicolor* and in *S. lividans*

REDIRECT PCR-targeting technology [26] was used to replace the genes of the entire *yefM/yoeB* operon to an apramycin (*aac(3)IV* gene) resistance cassette in *S. coelicolor* M145 and in *S. lividans* 1326. Mutagenic cassettes were flanked by the recognition sequence of yeast Flipase (FRT) and contained the *oriT* (FRT-*aac(3)IV-oriT*-FRT) conjugation transfer origin and were amplified with primers LS-013 and LS-014 (Table 1), using plasmid pIJ773 as template [26]. The cassettes generated were introduced into *E. coli* BW25113 (pIJ790), harboring the cosmid 7B11, and preinduced for λRed functions by the addition of arabinose to obtain a TA-disrupted version of the mutant cosmid. The disrupted cosmid, confirmed by restriction analysis, was isolated and transferred from *E. coli* ET12567 (pUZ8002) to *S. coelicolor* M145 and to *S. lividans* by conjugation. Exconjugants were selected on MSA medium containing apramycin (50 µg/mL), and the double crossover products were identified by their sensitivity to kanamycin (50 µg/mL). The antibiotic-resistant marker and the *oriT* region were eliminated in two steps. In the first, the corresponding disrupted cosmid was introduced into the *E. coli* DH5α (pBT30) strain (harboring the flipase gene, FLP), in which the recombination between both FRT mutagenesis cassette-flanking regions takes place. In this new cosmid, only 81 base pairs (SCAR) in-frame with the adjacent ORFs remained. Then, the SCAR cosmid was transferred to the *Streptomyces* apramycin resistance mutant strains by protoplast transformation, first selecting neomycin resistance clones (unique recombination), and then the apramycin- and neomycin-sensitive strains (double recombination). PCR assays confirmed the correct recombination in the new *Streptomyces* mutant strains.

**Table 1:** Oligonucleotides used.

Name	Sequence 5′-3′	Use
LS-001	TTTTTTGAATTCTGTGCGGCTGCCCTTCCGCC	Forward oligonucleotide to amplify the promoter of *SCO1968*. The sequence recognized by EcoRI is underlined.
LS-002	TTTTTTCATATGCGTACTCCTCGCGTCGAACG	Reverse oligonucleotide to amplify the promoter of *SCO1968*. The sequence recognized by NdeI is underlined.
LS-005	TTTTTTCATATGTCCATCACCGCCAGCGAAG	Forward oligonucleotide for cloning the TA operon into pXHis1 and pET22b. The sequence recognized by NdeI is underlined.
LS-008	TTTTTTCATATGAGGATCACTTTCACGTCCCAC	Forward oligonucleotide for cloning the toxin gene into pXHis1. The sequence recognized by NdeI is underlined.
LS-009	TTTTTTCTCGAGTCAGTAGTGGTAGCGCGCCTGG	Reverse oligonucleotide for cloning the toxin gene and TA operon into pXHis1. The sequence recognized by XhoI is underlined.
LS-013	CAGACTCGTACGATATCTTGTACCAGCCGAGGAAGGGAGGCACTGGTATGATTCCGGGGATCCGTCGACC	Forward oligonucleotide to obtain the mutagenic cassette. The sequence matching the sequence of the disruption cassette is underlined.
LS-014	GCTTCGGCTTTCGCCGGTCGCGGGTGTCGTGTCCGTACCGGCGGGTGTCATGTAGGCTGGAGCTGCTTC	Reverse oligonucleotide to obtain the mutagenic cassette. The sequence matching the sequence of the disruption cassette is underlined.
LS-019	TTTTTTGAATTCTCGGCCTCCTGTCGGGCTG	Forward oligonucleotide to amplify TA promoter. The sequence recognized by EcoRI is underlined.
LS-020	TTTTTTCATATGACCAGTGCCTCCCTTCCTCGG	Reverse oligonucleotide to amplify TA promoter. The sequence recognized by NdeI is underlined.
LS-021	TTTTTTCTCGAGGTAGTGGTAGCGCGCCTGGAC	Reverse oligonucleotide for cloning the TA operon into pET22b. The sequence recognized by XhoI is underlined.
LS-022	TTTTTTCTCGAGCGCCCGCTCCGCGTCCGGG	Reverse oligonucleotide for cloning the antitoxin gene into pET22b. The sequence recognized by XhoI is underlined.
AY-147	TAGAACACGGGTCCGACAGTCC	Forward oligonucleotide to amplify the promoter of *SCO1715*.
AY-148	ATCGCTCCCTCGCAACCGATTC	Reverse oligonucleotide to amplify the promoter of *SCO1715*.
AY-159	AGAGAGTATGTCCTAAATGTCCGG	Forward oligonucleotide to amplify the promoter of *SCO1480*.
AY-160	GCCTACGTCACCTCGGATGTCG	Reverse oligonucleotide to amplify the promoter of *SCO1480*.

### Toxicity evaluation in *E. coli*

*yoeBsl* DNA was amplified by PCR from *S. lividans* 1326 genomic DNA using primers LS-008 and LS-009 (Table 1). The resulting fragment was digested with NdeI and XhoI and ligated into plasmid pXHis1 [28] (Table 2) digested with the same enzymes to obtain plasmid pXHis-Tox, which was used as an intermediate plasmid. Plasmid pFUS2-Tox was obtained by digesting pXHis-Tox with NdeI and HindIII and ligated into plasmid pFUS2 [16] (Table 2), digested with the same enzymes. This construction placed the ORF of the putative toxin gene under the control of the arabinose-inducible promoter P_BAD_ and had the *fdt* transcriptional terminator at the 3′ end.

**Table 2:** Plasmids used.

Plasmid	Characteristics	Reference
**pIJ773**	pBluescript SK derivative containing the Apramycin resistance cassette.	[26]
**pXHis1**	pBluescript SK derivative. Ampicillin resistance. The *xysA* promoter from *S. halstedii* controls *xys1Δ* expression.	[28]
**pXHis-Tox**	pXHis1 derivative. The *xysA* promoter from *S. halstedii* controls toxin expression.	This work
**pXHis-TA**	pXHis1 derivative. The *xysA* promoter from *S. halstedii* controls TA expression.	This work
**pFUS2**	*E. coli* expression vector. Kanamycin resistance. P_BAD_ promoter.	[16]
**pFUS2-Tox**	pFUS2 derivative. P_BAD_ promoter controls toxin expression.	This work
**pFUS2-TA**	pFUS2 derivative. P_BAD_ promoter controls TA expression.	This work
**pN702GEM3**	*E.coli*/*Streptomyces* shuttle vector. Neomycin resistance. High-copy number.	[29]
**pN702Gem3-Tox**	pN702GEM3 derivative. The *xysA* promoter from *S. halstedii* controls toxin expression.	This work
**pN702Gem3-TA**	pN702GEM3 derivative. The *xysA* promoter from *S. halstedii* controls TA expression.	This work
**pKC796**	*E.coli*/*Streptomyces* shuttle vector. Apramycin resistance. Integrative plasmid.	[30]
**pKC796-Tox**	pKC796 derivative. The *xysA* promoter from *S. halstedii* controls toxin expression.	This work
**pKC796-TA**	pKC796 derivative. The *xysA* promoter from *S. halstedii* controls TA expression.	This work
**pET22b**	*E. coli* expression vector. Ampicillin resistance.	Novagen
**pET22b-Anti**	pET22b derivative. Expressing antitoxin gene with a His_6_ tag at the carboxy terminal.	This work
**pET22b-TA**	pET22b derivative. Expressing the TA operon. In this construction the toxin gene has a His_6_ tag at the carboxy terminal.	This work
**pGM160**	*E.coli*/*Streptomyces* shuttle vector. Thiostrepton and gentamicin resistance.	[31]
**pGM160-YefMsl**	pGM160 derivate. The *xysA* promoter from *S. halstedii* controls YefMsl expression.	This work

A similar strategy was used to amplify the genes of the complete operon (*yefM/yoeBsl)*, using primers LS-005 and LS-009 (Table 1). The resulting PCR fragment was digested with NdeI and XhoI and ligated into plasmid pXHis1 digested with the same enzymes to obtain plasmid pXHis-TA, which was used as an intermediate plasmid. Plasmid pFUS2-TA was obtained by digesting pXHis-TA with NdeI and HindIII and ligated into plasmid pFUS2 digested with the same enzymes. This construction placed the complete operon under the control of the arabinose-inducible promoter P_BAD_ and had the *fdt* transcriptional terminator.

*E. coli* SC36 cells transformed with pFUS2 (control), pFUS2-Tox or pFUS2-TA were grown at 37°C on LB broth supplemented with 50 µg/mL of kanamycin to an OD_600_ of 0.5–0.8 and the cultures were divided into two parts. One half of each culture was grown in the presence of 0.2% glucose (repression conditions) and the other half in the presence of 0.2% arabinose (induction conditions). Culture growth was monitored measuring the OD_600_. In addition, samples were obtained 1 hour after induction and 5-µl drops of different dilutions of cultures were spread onto the surface of LB agar supplemented with 50 µg/mL of kanamycin. Plates were incubated overnight at 37°C.

### Toxicity evaluation in *Streptomyces*

Multicopy plasmids were generated by cloning the *yoeBsl* toxin encoding gene or the complete operon into plasmid pN702Gem3 [29] (Table 1). Plasmids pXHis-Tox and pXHis-TA were digested with BglII and the DNA fragments containing the genes (Tox and TA) were ligated with pN702Gem3 (Table 2) digested with the same enzyme. The toxin and antitoxin-toxin genes of the resulting plasmids, pN702Gem3-Tox and pN702Gem3-TA, are regulated by the xylanase promoter *xysA*p [17]. All the constructions were flanked by two transcriptional terminators.

Integrative *Streptomyces* plasmids, whose gene expression was regulated by *xysA*p, were generated by cloning the corresponding BglII/BglII fragments into the integrative plasmid pKC796 [30] (Table 2). Plasmids pKC796-Tox and pKC796-TA were obtained respectively.

Multicopy plasmid pGM160-YefMsl was generated by cloning the antitoxin gene between BstBI and BamHI sites of pGM160 plasmid [31].

These plasmids were introduced by protoplast transformation into *S. lividans* 1326 wt and ΔTA (lacking the chromosomal copy of the system) and in *S. coelicolor* M145 wt and the corresponding ΔTA mutant. Cell viability was estimated by checking their growth in R2YE medium after incubation at 30°C.

### Protein purification

*S. lividans* antitoxin-encoding DNA (*yefMsl*) and the DNA corresponding to the TA operon (*yefM/yoeBsl*) were amplified by PCR using primers LS005/LS022 and LS005/LS021 respectively (Table 1) and cloned between the NdeI and XhoI sites in the pET-22b vector (Novagen) to produce the antitoxin or toxin, respectively, tagged C-terminally with a hexahistidine motif, yielding pET22b-Anti and pET22b-TA respectively.

YefM-His_6_sl and the YefM/YoeB-His_6_sl complex were overproduced in *E. coli* BL21 (DE3) transformed with the corresponding plasmids. Five hours after induction with 1 mM IPTG, cells were harvested at 5000×*g* at 4°C for 10 min. The cell pellet was resuspended in lysis buffer (5 mM Na_2_HPO_4_/NaH_2_PO_4_, pH 7.5, 300 mM NaCl, 0.1%Triton X100, 5 mM imidazole), and then sonicated and centrifuged for 30 min. at 100.000×*g*. The supernatant was applied to a column containing 2 ml of NTA-Ni resin (Qiagen). The column was washed three times with 5 mL of washing buffer 1 (5 mM Na_2_HPO_4_/NaH_2_PO_4_, pH 7.5, 300 mM NaCl, 0.1% Triton X100, 20 mM imidazole) and twice with 5 mL of washing buffer 2 (5 mM Na_2_HPO_4_/NaH_2_PO_4_, pH 7.5, 300 mM NaCl, 0.1% Triton X100, 30 mM imidazole). Tagged proteins were eluted 3 times with 0.5 mL of elution buffer 1 (5 mM Na_2_HPO_4_/NaH_2_PO_4_, pH 7.5, 300 mM NaCl, 0.1% Triton X100, 250 mM imidazole) and twice with elution buffer 2 (5 mM Na_2_HPO_4_/NaH_2_PO_4_, pH 7.5, 300 mM NaCl, 0.1% Triton X100, 1 M imidazole). Fractions containing the highest concentrations of proteins were pooled and dialyzed with *D-Tube TM Dialyzer Maxi* (Novagen) for 48 h against 2 L of dialysis buffer (50 mM Tris, pH 7.5, 100 mM NaCl, 10% glycerol).

Purification of the toxin YoeB-His_6_sl was carried out in *E. coli* BL21 (DE3) cells transformed with pET22b-TA that produces the complex toxin-antitoxin. Protein production and purification were done as described previously but the second wash of the NTA-Ni resin, containing the complex antitoxin/toxin-His_6_, was performed with 5 mM Na_2_HPO_4_/NaH_2_PO_4_, pH 7.5, 300 mM NaCl, 0.1 Triton X100%, 20 mM imidazole, 6 M guanidine hydrochloride to denature the antitoxin protein YefMsl [21]. YoeB-His_6_sl was then eluted as described above. Fractions containing the highest concentrations of protein were pooled and dialyzed successively for 2h against 500 mL of dialysis buffer containing 3 M, 2 M, and 1 M urea, followed by 24 h against 2 L of dialysis buffer without urea. The concentration of TA complex was estimated assuming an antitoxin∶toxin ratio of 2∶1), as has been described for *E. coli* YefM-YoeB complexes [14]).

### Electrophoretic mobility assays (EMSA)

The TA promoter was amplified by PCR from *S. lividans* 1326 genomic DNA using primers LS-019 and LS-020 (Table 1). The resulting PCR fragment (196 bp) was used in the binding reactions with the proteins. Different *Streptomyces* promoters unrelated to TA systems were used as controls: namely, the promoter of SCO1968 (a putative secreted hydrolase, amplified with primers LS-001 and LS-002, Table 1); SCO1715 (a putative homogentisate 1,2-dioxygenase, amplified with primers AY-147 and AY-148, Table 1), and SCO1480 a hypothetical protein, amplified with primers AY-159 and AY-160, Table 1).

The binding reactions contained 150–200 ng of DNA, 10 mM Tris-HCl, pH 7.5, 10 mM MgCl_2_, 100 mM NaCl, 10% glycerol, 2 µg of salmon sperm DNA, and different concentrations of the proteins. The mixtures were incubated at 30°C for 20 min and electrophoresed at 4°C on 5% native polyacrylamide gels in TBE buffer 1× (90 mM Tris, 89 mM boric acid, 2 mM EDTA, pH 8.3). DNA was visualized by gel staining with ethidium bromide (0.5 µg/mL in TBE buffer). When YefM-His_6_sl and YoeB-His_6_sl were added separately, the two proteins were first incubated at incubation at 30°C for 20 min prior to the addition of DNA.

### Assay of protein synthesis *in vitro*

Cell-free protein synthesis was performed with an *E. coli* T7 S30 Extract System for Circular DNA (Promega). The reaction mixture was prepared as described in the manufacture's protocol. Then, different amounts of YoeB-His_6_sl and YefM-His_6_sl were added in a final volume of 29 µl. The reaction was started by the addition of pET11a-*mazG* plasmid DNA [15], [32] and the mixture was incubated for 30 min at 37°C. Proteins were precipitated with acetone and analyzed by 15% SDS-PAGE. The dried gel was followed by autoradiography.

### Primer extension analysis *in vivo*

For primer extension analysis of mRNA cleavage sites *in vivo*, total RNA was extracted from the *E. coli* BL21(DE3) cells containing pFUS2-Tox at different time points after YoeBsl induction. Primer extension was carried out as described previously [33].

### Sequence analysis

All constructions were sequenced in both strands using a Perkin Elmer ABI Prism 377 DNA sequencer. *In silico* plasmids were obtained with the Gene Construction Kit software (GCK, Textco). BPROM software (http://linux1.softberry.com/berry.phtml) was used to search for conserved sequences in the putative promoter of the TA system.
